# Supplementary material for: Complexome profiling of the Chlamydomonas psb28 mutant reveals TEF5 as an early PSII assembly factor
Source: Plant Cell. 2025 Mar 17;37(6):koaf055. doi: 10.1093/plcell/koaf055 (PMC12164590; doi:10.1093/plcell/koaf055)
Supplement: koaf055_Supplementary_Data [file koaf055_supplementary_data.zip › psb28_interactive.zip!/plots/intensity/qualityPlots/peptides/CAH3_Cre09.g415700.t1.2.html]

### Cre09.g415700.t1.2

Trivial name: CAH3  
SeqCoverage [median]: 19.40  
Mass: 33.46 kDa  
  
 

| Sequence | MissedCleavage | Mass [Da] | wtIntensity | muIntensity | Score |
| --- | --- | --- | --- | --- | --- |
| QSPINIPLNTSAPK | 0 | 1478.81 | 0.00 | 2137.05 | 76.93 |
| TYATNTRPLQLLNSR | 1 | 1746.94 | 3574.83 | 7295.00 | 59.19 |
| YAMEAHLVHK | 0 | 1197.60 | 681.69 | 2770.63 | 85.29 |
|  |  |  | 4256.5 | 12202.7 |  |
